# The Institutional Library

**Published:** 1921-06-25

**Authors:** 


### THE INSTITUTIONAL LIBRARY.

**Diseases of the Throat, Nose, and Ear.** By DAN McKENZIE, M.D., F.R.C.S. (London: Wm. Heinemann; 1920. Pp. 646. Price 42s. net.)

THIS new text-book, by a well-known laryngologist, is a definite acquisition to the literature of the specialty with which it deals. It is not exhaustive over such matters as the details of operative technique in complicated major operations; but it covers the ground pretty fully in a practical, common-sense way with regard to diagnosis and treatment. That is to say, it is the man taking up this branch of surgery, rather than the man who has been devoting himself to it for years, for whom it is best suited and (no doubt), in fact, designed.

On reading through it there is singularly little to be found, for a text-book of this scope, which will not commend itself to those who have kept abreast with modern advances in the science of oto-laryngology. No reference is made, apparently, to the value of intratracheal anæsthesia in facilitating some of the more difficult operations about the air-passages. In describing Vincent's angina, there is no reference to the associated stomatitis and gingivitis as an aid to diagnosis in doubtful cases. In connection with diphtheritic paralysis, it is remarked that ocular paralysis may be overlooked; surely "ocular" should be qualified by a preceding "intrinsic." Mr. Dan McKenzie has produced a text-book of remarkable merit. It is especially pleasing to notice that he is thoroughly alive to the virtues of diathermy in suitable cases; there is still a strange reluctance in some London clinics to employ this valuable measure as freely and fully as it ought to be employed.

**Transactions of the National Association for the Prevention of Tuberculosis.** Eighth Annual Conference, Liverpool, October 1920. Pp. 234. (London: Adlard & Son. 1920.)

IT is easy to talk about tuberculosis, easy nowadays for anyone. This truth is sufficiently proved by the quarto report before us. But it is hard to talk to good purpose. The medical side of prophylaxis has been exhaustively set out for so many years now that it is doubtful whether much can be done by more repetition. The great fundamental that the social and economic side is of vastly greater importance unfortunately knocks the bottom out of so many arguments confidently put forward. It stultifies Professor Moore's recipe of a well-equipped research commission, or the parliamentary doctor's vague plea for a strengthening of the hands of the Health Minister. It completes the easily achieved confusion of the dairy crank—at least one must apply that term to the gentleman who promised to stop any further tuberculous infection of school children in three weeks by looking to the country's milch cattle. It renders nugatory the surely desperate claim that "existing anti-tuberculosis machinery" can, if worked with a will, bring about the huge results it is claimed capable of. So long as the profession, and indeed the tuberculosis specialty, boggles at this elementary fact, so long will both, consciously or unconsciously, be sinning against the light, and so long will such conferences as that here reported be wasting three-parts of their efforts. We hold no brief for feminists, but still it is but fair to mention that the only bold recognition of the above truth came from a woman, and a lay woman at that. Councillor Emily Palmer in her bold speech went straight to the political and economic aspects of the matter—committing no breach of decorum thereby, for political allusions in a contrary sense were freely made by other speakers. "Out of the mouths . . ."!

**Injuries of the Peripheral Nerves.** By H. S. SOUTTAR, C.B.E., F.R.C.S., and EDWARD W. TWINING, M.R.C.S., L.R.C.P. (Bristol: John Wright and Sons, Ltd. London: Simpkin, Marshall and Co., Ltd. Pp. 152. Price 18s. 6d. net.)

THIS is emphatically a sound book. The authors insist upon both a clear apprehension of the physiological principles underlying the structure, degeneration, and repair of nerves and muscle, and also a minutely accurate knowledge of anatomy as indispensable adjuncts to the surgeon's armoury of weapons in this delicate and complex field of surgery. In war injuries, from which much of the writers' knowledge and experience have been gained, the fact that ordinary anatomical niceties are, more often than not, obliterated by shell and sepsis, does but render knowledge of normal anatomy all the more important, and the surgeon's ingenuity and resource in dealing with torn and destroyed nerve and muscle must be subservient to the great basal physiological principles which determine the life history of these tissues. Such knowledge will impress upon the operator the necessity for precision in suturing nerve, make clear the futility of attempting to bridge gaps by other than autogenous grafts, and cause him to make sure he has a living primitive sheath to deal with before he expects or attempts recovery of a divided nerve. The anatomical relations of the various nerves is described accurately and clearly, and the electrical reactions also are adequately dealt with. On this foundation is built up a treatise which marks a distinct advance in this branch of surgery. Of the whirlpool bath the authors write enthusiastically, although some have rather doubted its utility.

**Some Conclusions on Cancer.** By CHARLES CREIGHTON, M.D. (London: Williams & Norgate. 1920. 114 illustrations. Pp. 336. Price 42s.)

THIS book represents the result of over fifty years' close work upon the much-discussed ætiology of cancer. The arguments put forward in support of these new theories must be beyond the comprehension of many of us; nor are they supported by the trend of the most recent investigations.

The author traces the onset of cancer to many causes. Many of the views put forward are highly controversial and differ widely from those generally accepted to-day; as an example we have the following on page 55: ". . . the blood of the fœtus, effused accidentally in puncturing the membranes, can act septically on the uterine tissues and juices so as to produce infective thrombosis and general septicæmia."

